# Supplementary figures and images for: Economic impact and clinical benefits of clinical pharmacy interventions: A six-year multi-center study using an innovative medication management tool
Source: PLoS One. 2025 Jan 17;20(1):e0311707. doi: 10.1371/journal.pone.0311707 (PMC11741631; doi:10.1371/journal.pone.0311707)

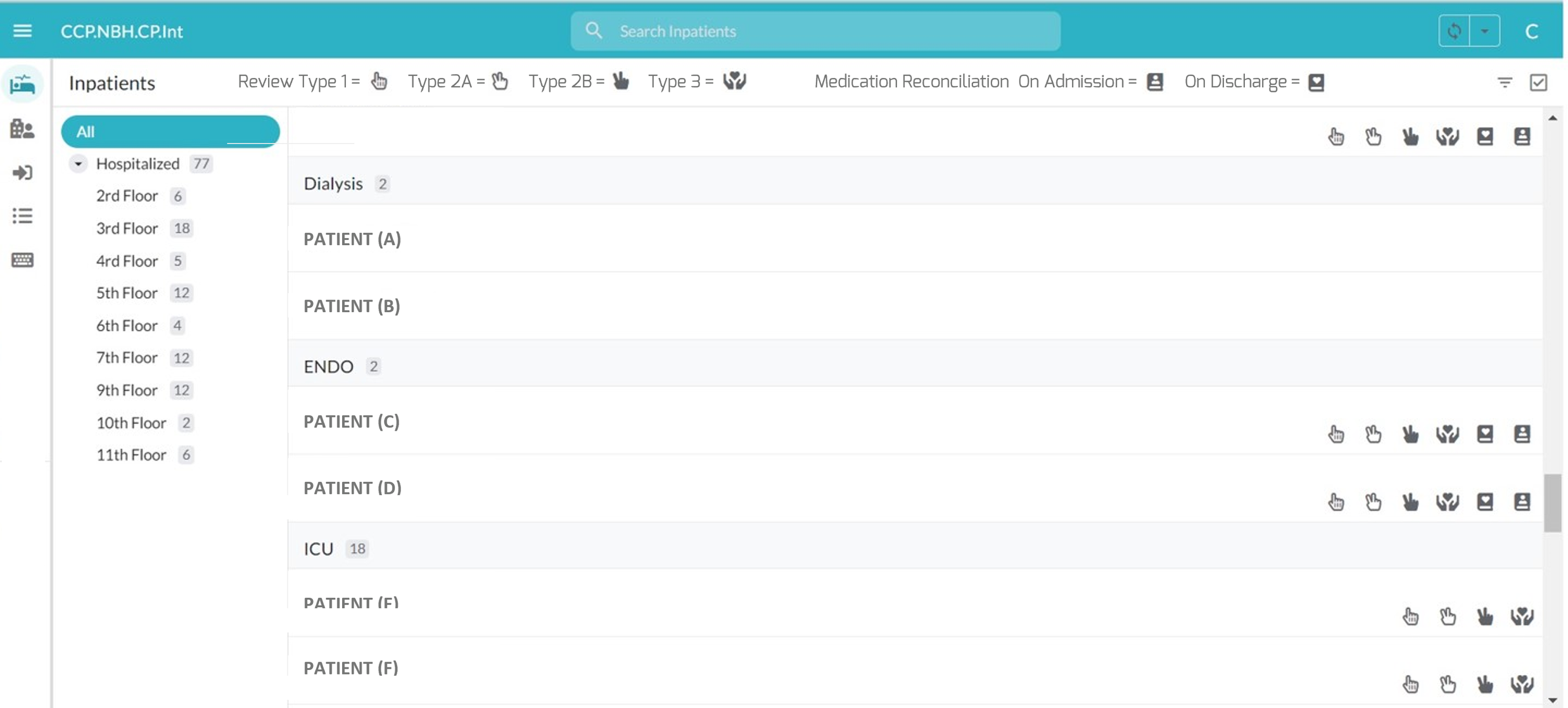

Supplement: S1 Fig — (TIF) [file pone.0311707.s001.tif]

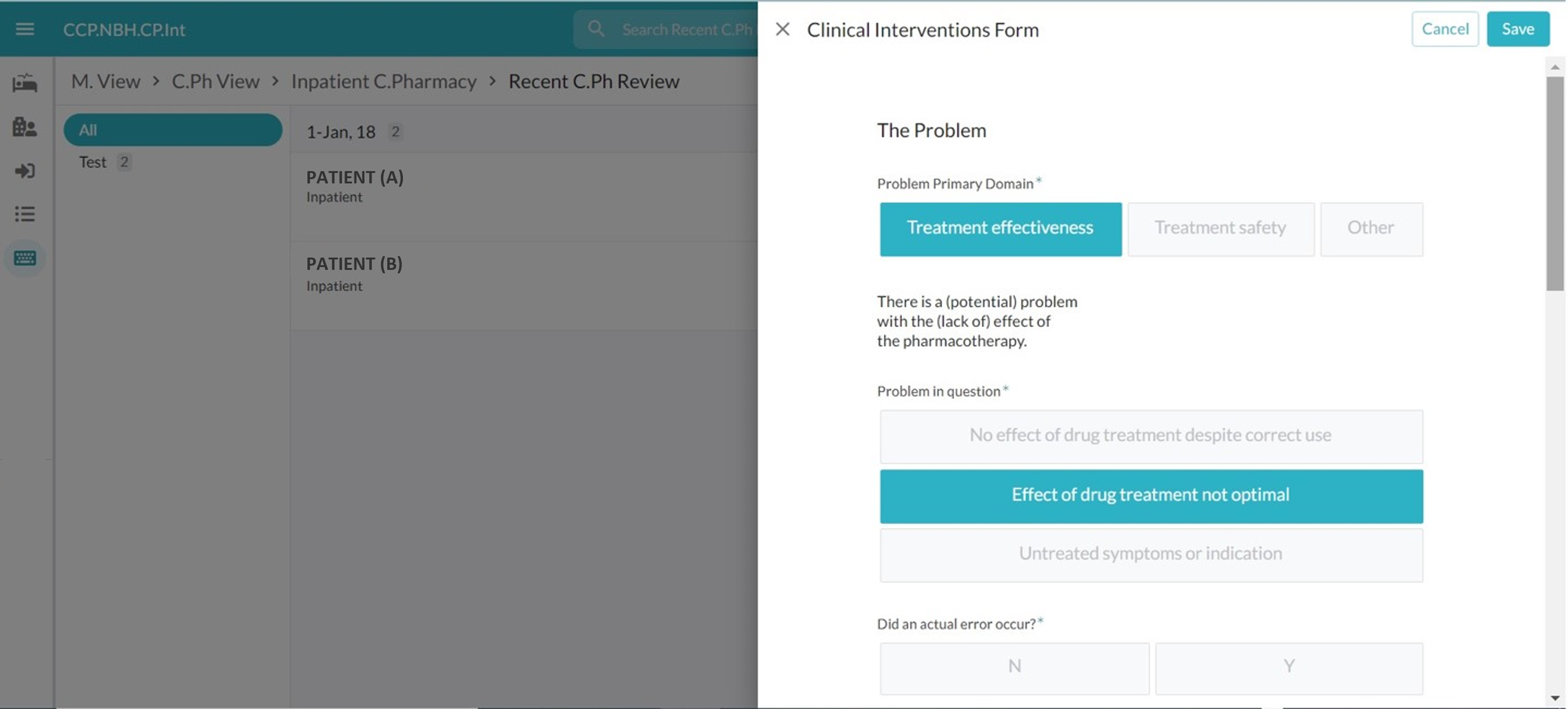

Supplement: S2 Fig — (TIF) [file pone.0311707.s002.tif]
