# Supplementary material for: Economic impact and clinical benefits of clinical pharmacy interventions: A six-year multi-center study using an innovative medication management tool
Source: PLoS One. 2025 Jan 17;20(1):e0311707. doi: 10.1371/journal.pone.0311707 (PMC11741631; doi:10.1371/journal.pone.0311707)
Supplement: S1 File — (PDF) [file pone.0311707.s003.pdf]

S1 File: Expert Panel Composition and Expertise

The cCON.cleo panel method was developed with contributions from a team of seasoned professionals, each bringing expertise from their respective fields. This collaborative effort included:

- An **Intensivist** with over two decades of experience in critical care, certified with a fellowship from the Egyptian Board in Critical Care. This professional's insights into critical patient care added depth to our approach to managing complex cases.
- A **Cardiologist** with an MD and 11 years of experience in cardiology, provided valuable cardiovascular knowledge to the discussions.
- A **Neurologist**, holding an MD and 13 years of experience in neurology, contributed expertise on neurological aspects relevant to DTP management.
- An **Internist** with an MD and 26 years of experience in internal medicine, offering a comprehensive perspective on the interplay between various internal conditions and DTPs.
- A **Clinical Pharmacist** with a PharmD, a fellowship from the Egyptian Board of Clinical Pharmacy, and 12 years of experience in pharmaceutical care and medication therapy management, bringing a crucial perspective on pharmacological considerations.

This group's diverse expertise was instrumental in formulating the cCON.cleo method. The method was informed by their collective experiences, CHG's 2023 data on average hospital stays following DTP incidents, and literature highlighting the potential extension of hospitalization due to DTPs. The consensus reached on hospital stay extensions for various levels of DTP consequences is detailed in Table 1.
