# Supplementary material for: Economic impact and clinical benefits of clinical pharmacy interventions: A six-year multi-center study using an innovative medication management tool
Source: PLoS One. 2025 Jan 17;20(1):e0311707. doi: 10.1371/journal.pone.0311707 (PMC11741631; doi:10.1371/journal.pone.0311707)
Supplement: S2 File — (PDF) [file pone.0311707.s004.pdf]

S2 File: Detailed Direct Cost Savings (DCS) Calculations

The Direct Cost Savings (DCS) is calculated based on the nature of the recommended intervention as the following:

a) Adding a new Concerned Medication:

$$\mathrm{DCS} = -1 \times [\mathrm{Concerned\ Medication\ Cost\ (CMC)}]$$

, indicating a non-favorable cost.

b) Modifying a Concerned medication's form, dose, duration, or a change to a different medication.

$$\mathrm{DCS} = [\mathrm{Concerned\ Medication\ Cost\ (CMC)}] - [\mathrm{Modefied\ Medication\ Cost\ (MMC)}]$$

c) Discontinuation of Concerned Medication

$$\mathrm{DCS} = [\mathrm{Concerned\ Medication\ Cost\ (CMC)}]$$

, resulting in favorable costs.

d) Requesting other non-drug services such as monitoring costs,

$$\mathrm{DCS} = -1 \times [\mathrm{Other\ Non-Drug\ Requests\ Cost\ (ONDRC)}]$$

e) Interventions that do not necessitate direct cost savings (e.g., added medication instruction)

$$\mathrm{DCS} = 0.$$

All direct cost savings (DCS) variables in these equations are rooted in data from 2023, encompassing medication, services, and full-time equivalent (FTE) costs. Within this framework,

a) Concerned Medication Cost (CMC) determined as

$$[\mathrm{Concerned}\ Medication\ Acquisition\ Cost\ (CMAC)] + [Concerned\ Medication\ Labor\ Cost\ (CMLC)]$$

i. Concerned Medication Acquisition Cost (CMAC) calculated as

$$CMAC = [Number\ of\ unit\ doses] \times [Unit\ Dose\ Cost] \times [Frequency\ Per\ Day] \times [Duration\ in\ Days]$$

ii. Concerned Medication Labor Cost (CMLC) calculated as

$$CMLC = [Average\ Cost\ of\ Dispensed\ One\ Medication\ Order] \times [Number\ Dispensing\ Per\ Day] \times [Duration\ in\ Days]$$

The Average Cost of Dispensed Medication One Order was set at 1.77 (EGP) sourced from the hospital's Enterprise Resource Planning (ERP) system and Full-Time Equivalent (FTE) rate. The dispensing frequency is consistently defined as 1, reflecting the hospital's practice of dispensing doses to the care units at 24-hours intervals.

b) Modified Medication Cost (MMC) follows a similar structure.

c) The Other Non-Drug Recommendation Cost (ONDRC) is based on hospital cost center estimates for services and procedures for the fiscal year 2023.
