# Supplementary material for: Economic impact and clinical benefits of clinical pharmacy interventions: A six-year multi-center study using an innovative medication management tool
Source: PLoS One. 2025 Jan 17;20(1):e0311707. doi: 10.1371/journal.pone.0311707 (PMC11741631; doi:10.1371/journal.pone.0311707)
Supplement: S3 File — (PDF) [file pone.0311707.s005.pdf]

S3 File: Example of cost-avoidance analysis of a Clinical Pharmacy Intervention

Case Overview: A clinical pharmacist reviewed a 72-year-old female patient in the CCU who was diagnosed with atrial fibrillation 3 days prior. The patient's CHA2DS2-VASc score was 3. The intervention involved initiating Metoprolol extended release (50 mg once daily) for long-term rate control without cardioversion and recommending Apixaban (5 mg twice daily), as no anticoagulant was initially prescribed.

Note: The reported opinion of the expert panel suggests that the likelihood of an adverse consequence (thromboembolic stroke), is possible, with the consequence level assessed at 4, indicating major consequences.

Calculation of the Economic Impact:

1. Probabilities: Probability of Consequence (pCON) = 0.1 (based on expert opinion); Probability of Trajectory Change (pTC) = 1.
2. Cost of consequence (cCON): Estimated at EGP 73,500 calculated from a daily cost of EGP 21,000 over 3.5 days.
3. Direct Cost Saving (DCS):
   a. Concerned Medication Acquisition Cost (CMAC) = EGP 193.375 (calculated as 1 dose × EGP 13.8125 per dose × 2 doses per day × 7 days).
   b. Concerned Medication Labor Cost (CMLC) = EGP 19.107 (calculated as EGP 2.72964 per medication order × 1 dispensing per day × 7 days).
   c. Total Concerned Medication Cost (CMC) = EGP 212.482 (sum of CMAC and CMLC).
   d. DCS is -EGP 212.482, as this reflects an added cost due to the new medication.
4. Intervention Factor (iFactor): 1 (as the intervention was accepted).
5. Success Factor (sFactor): 1 (indicating a resolved problem).
6. Cost of pharmacist (cPharm): EGP 245.

Cost-Avoidance Calculation: [pTC×({pCON×cCON}+DCS)×iFactor×sFactor]-[cPharm]

Cost- Avoidance Calculation: [1 × ({0.1 × 73,500} + (- 212.482)) × 1 × 1] - [245] = EGP 6,892.518
