# Supplementary material for: Economic impact and clinical benefits of clinical pharmacy interventions: A six-year multi-center study using an innovative medication management tool
Source: PLoS One. 2025 Jan 17;20(1):e0311707. doi: 10.1371/journal.pone.0311707 (PMC11741631; doi:10.1371/journal.pone.0311707)
Supplement: S4 File — (PDF) [file pone.0311707.s006.pdf]

CLINICAL PHARMACY PRACTICE DASHBOARD

RFA	COVERAGE	PATIENTS	Reviews	R/Patient	INT	Cost Avoidance	C.Ph ROI
100%	75.2%	3.1K	7.1K	2.3	0.4K	2.86M	7.279

Print

Review Date
01-Jul-23 to 31-Oc..

Coverage
Multiple values

Appropriateness Review Vs TYPE III Comprehensive Review

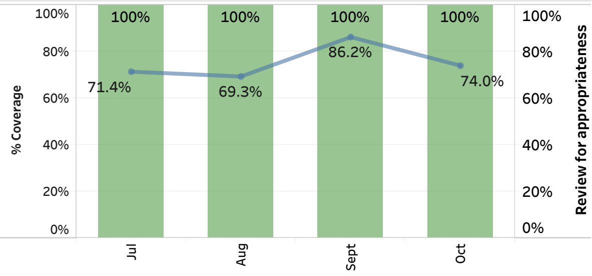

This chart displays a **100%** appropriateness review of all medications ordered, along with an average clinical pharmacy comprehensive (Type III) coverage rate of **69% to 86%** for hospital patients.

Sites Coverage

Room N° (group)	Jul	Aug	Sept	Oct	Grand Total
2nd Floor	84%	82%	92%	80%	90%
3th Floor	91%	88%	87%	94%	94%
4th Floor	85%	79%	93%	86%	86%
5th Floor	93%	94%	99%	98%	88%
CCUs	100%	100%	100%	100%	100%
M-ICU	100%	100%	100%	100%	100%
NICU-PICU	100%	100%	100%	100%	100%
Post-Cath	67%	54%	80%	82%	68%
S-ICU	100%	100%			100%
Grand Total	87%	84%	86%	89%	91%

This chart illustrates **54% to 100%** comprehensive clinical pharmacy coverage (Type III) for patients in different hospital Sites

% Coverage
% Coverage
% Review for appropria..

% Site Coverage
54% 100%

Required Pharmacists Correlated to Patient Day

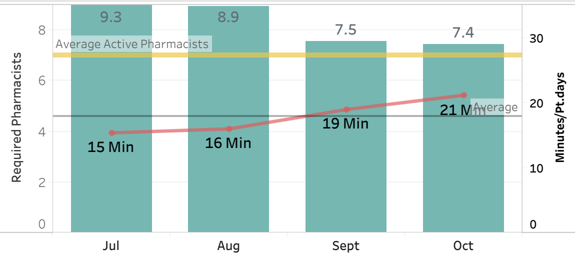

This chart compares the number of **7** active clinical pharmacists to the **7.4 to 9.3** standard requirement needed to cover patients. The calculation considers the average number of active clinical pharmacists, the number of adjusted patient days (excluding day cases and out-of-scope specialties), and **15 Min to 21 Min** dedicated minutes per patient day. A 12-hour shift should cover 12-14 critically ill or 30-36 non-critically ill patients, with 20-24 minutes per adjusted patient day.

Ass. Tasks insights

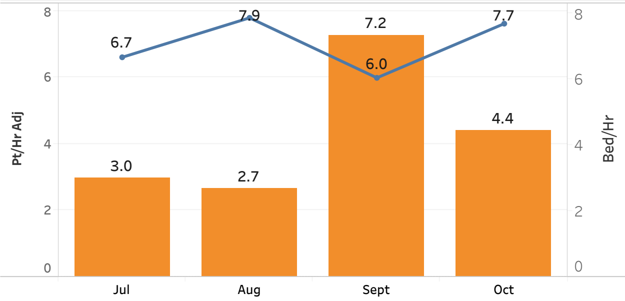

This chart shows that, on average, each active clinical pharmacist is assigned **6.0 to 7.9** beds per hour during a 12-hour shift and is responsible for an average of **2.7 to 7.2** patients per hour during the same shift.

Measure Names
Minutes/Pt.days
Required Pharmacists

Measure Names
Bed/Hr
Pt/Hr Adj

IHI Severity

	Not an Error<br>A	Near Miss<br>B	Reach Patient<br>C
Jul	48%	47%	5%
Aug	49%	47%	4%
Sept	50%	47%	3%
Oct	55%	40%	5%

This chart illustrates the percentage of drug-related problems as per the NCC MERP Index

Intervention / Reviewed Patient

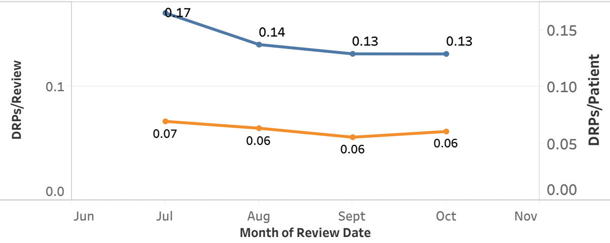

This chart displays an average of **0.13 to 0.17** clinical intervention(s) for each patient reviewed by the clinical pharmacists

% IHI of Total DRPs
3% 55%

Measure Names
DRPs/Pt
DRPs/Review

DRPs

	Jul	Aug	Sept	Oct
Effect of drug treatment not optimal	17%	30%	19%	43%
Adverse drug event (possibly) occurring	44%	27%	39%	25%
Unnecessary drug-treatment	13%	10%	5%	15%
Untreated symptoms or indication	20%	12%	23%	15%
Unclear problem/complaint	3%	18%	8%	
No effect of drug treatment despite correct use	2%	3%	6%	1%

This chart depicts the primary drug-related problem percentage for each month

Root Causes

		Jul	Aug	Sept	Oct
Prescribing	Condition without Treatm..	20%	20%	30%	22%
	No indication	13%	11%	7%	10%
	Dose too low	5%	10%	5%	9%
	Dose too high	17%	14%	13%	11%
	Inappropriate based on g..	7%	6%	4%	6%
	Dosage regimen too frequ..	8%	9%	11%	10%
	Duplication	9%	4%	6%	11%
	Dosage regimen not frequ..	8%	12%	10%	10%
	Inappropriate combination	7%	7%	5%	7%
	Duration too long			1%	
	Dose timing instructions ..	1%			
	Inappropriate form/formu..	1%	1%	3%	4%
Dispensing	Necessary information no..		1%		
Use	Drug administered via wr..			2%	
	Inappropriate timing of a..			1%	
Other	No or inappropriate outco..	2%	3%	1%	
	Adverse drug event	3%		1%	
	No obvious cause		2%	1%	

This chart represents the main root causes of drug-related problems for each month

Count of Problem Causes
1 34

% Clinical Interventions
1% 44%

Untreated Condition

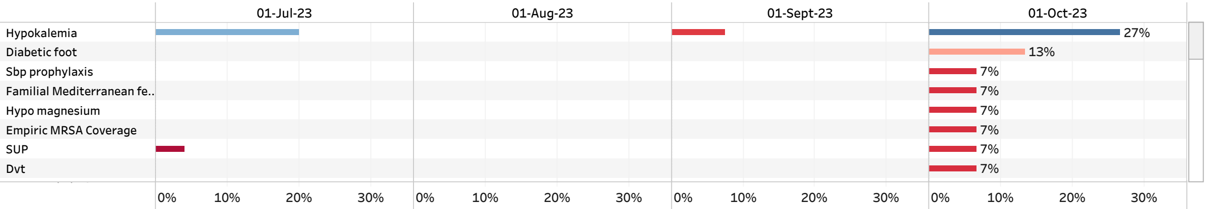

Untreated Conditions
3.70% 29.63%

This chart shows the top identified Untreated conditions between reviewed patients in each month

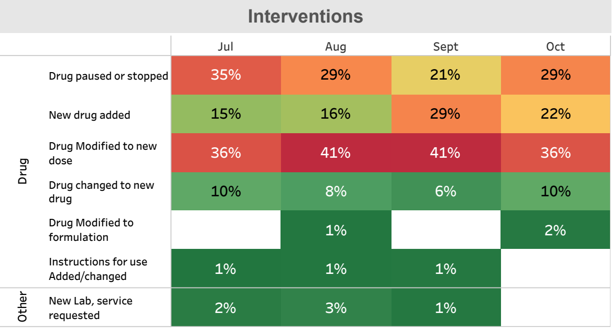

This chart visually presents the distribution of various interventions carried out by clinical pharmacists each month in terms of percentages

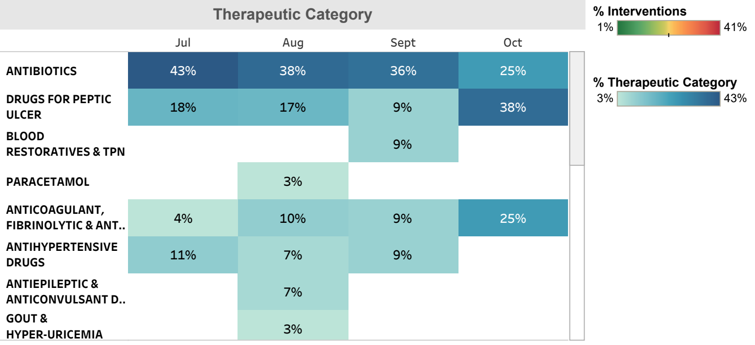

This chart visually depicts the percentage of identified drug-related problems categorized by therapeutic category

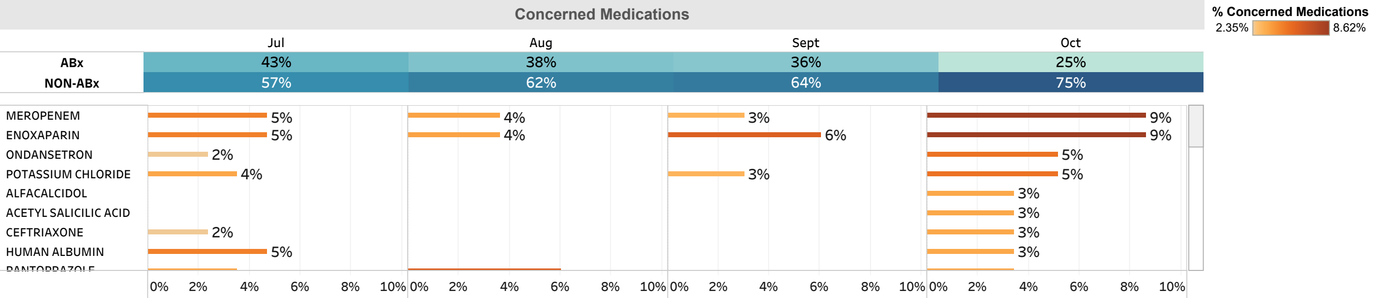

This chart provides a visual breakdown of the percentage of antibiotics versus non-antibiotics among the total identified drug-related problems. Additionally, it includes a detailed breakdown of concerned medication percentages within the total identified drug-related problems

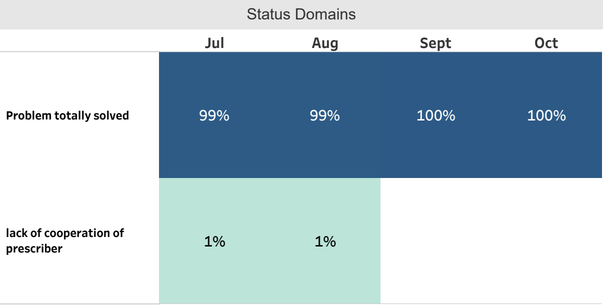

This chart displays the percentage of identified drug-related problems based on their final status after clinical pharmacy interventions

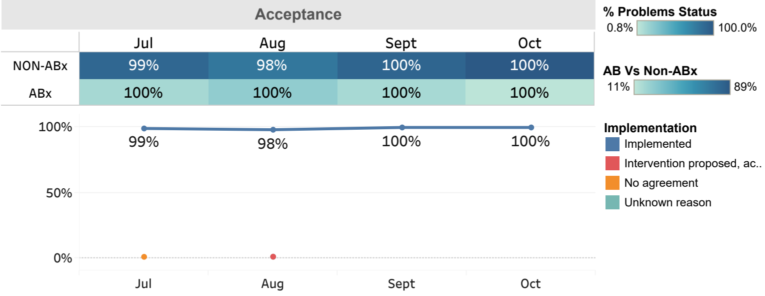

This chart visually breaks down the percentage of intervention acceptance by primary care providers (physicians). It also provides a detailed breakdown of acceptance percentages within antimicrobials versus non-antimicrobials among the total identified drug-related problems

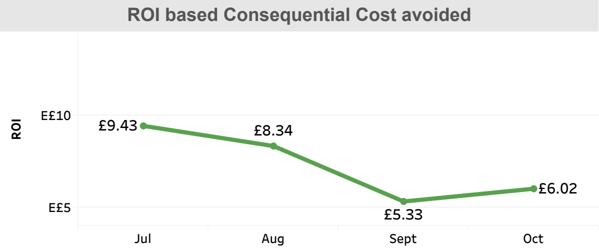

This chart demonstrates that each **1 E£** invested in pharmaceutical services, as per the current year's budget and FTE (Full-Time Equivalent) results, leads to the avoidance of **E£5 to E£9** consequential costs

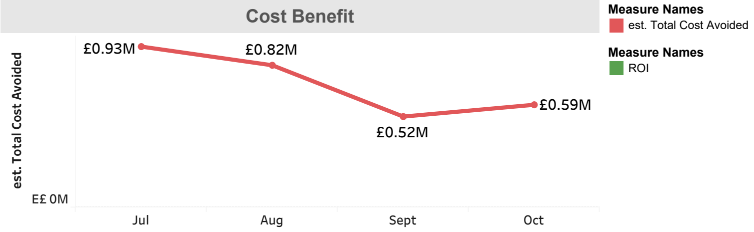

This chart illustrates a monthly estimated total Cost avoidance of **E£ 1M** achieved through clinical pharmacy services
