# Supplementary material for: Economic impact and clinical benefits of clinical pharmacy interventions: A six-year multi-center study using an innovative medication management tool
Source: PLoS One. 2025 Jan 17;20(1):e0311707. doi: 10.1371/journal.pone.0311707 (PMC11741631; doi:10.1371/journal.pone.0311707)
Supplement: Attachment — Submitted filename: Response to Reviewers.docx [file pone.0311707.s007.docx]

# Point – to – Point reply to reviewers’ comments.

We appreciate the valuable feedback provided by Reviewer #1, which has significantly contributed to enhancing the quality and clarity of our manuscript. Below, we address the comments point by point:

1. **Cumulative ROI is represented but a yearly analysis could derive more on the trends as per yearly basis.**

Thank you for the suggestion to include ROI trends on a yearly basis. While the current results section highlights the highest and lowest ROI values during the study period, we acknowledge that presenting a detailed yearly breakdown can provide additional insights. The yearly ROI trends are already depicted in Fig. 1, showcasing variations across the six years. We have updated the ROI results section to include the yearly ROI breakdown, and to enhance clarity, we explicitly mentioned that Fig. 1 provides a detailed depiction of the annual trends. This will ensure that readers can readily access and interpret the yearly variations in ROI.

2. **Incorporating a matched cohort or comparing data from hospitals without clinical pharmacy services could strengthen the conclusions. If a control group is not possible, stating this constraint more explicitly might be beneficial.**

Thank you for the suggestion to incorporate a matched cohort or comparison with data from hospitals without clinical pharmacy services to strengthen the conclusions. In the revised version, we have explicitly highlighted that including a control group was not feasible due to logistical challenges, such as the lack of standardized data from comparable hospitals, and ethical considerations, including the withholding of clinical pharmacy services that are integral to patient care. We have also clearly stated this constraint in the updated limitations section, ensuring transparency and aligning with your recommendation. The study, therefore, focuses on evaluating the pharmacoeconomic impact of clinical pharmacy interventions and assessing clinical benefits at the level of DTPs resolution. We appreciate your valuable feedback and have addressed this thoroughly in the revised manuscript.

3. **While the probability estimates used in cost-avoidance calculations are plausible, further information on their derivation and validation would improve the manuscript's clarity. It would be particularly useful to address any potential limitations of expert panel assessments in establishing probabilities.**

Thank you for your comment regarding the derivation and validation of the probability estimates used in the cost-avoidance calculations. The manuscript has been updated to clarify how the expert panel quantified and calculated the probabilities, including the use of the Nesbit probability scale for standardization. Additionally, the potential limitation of expert panel subjectivity has been explicitly addressed in the limitations section to ensure transparency.

4. **Consider explaining the ramifications of removing non-patient-specific interventions, as this may underestimate the true benefit of clinical pharmacy services.**

Thank you for your thoughtful comment regarding the exclusion of non-patient-specific activities. This study focuses on clinical pharmacist-led interventions, with the cost estimation model tailored to evaluate their unique contributions. The pTC (probability of trajectory change) parameter ensures the assessment remains specific to the direct impact of clinical pharmacists. We recognize that non-patient-specific activities, such as formulary management, ASP, and IV admixture services, are valuable components of clinical pharmacy services. However, these often involve multidisciplinary teams and were beyond the scope of this study. To address this, we have clarified in the limitations section that incorporating these activities could be explored in future studies with a broader scope, which would require a comprehensive cost-avoidance and ROI models to account for additional variables and economic factors. This study provides a precise evaluation of clinical pharmacist-led interventions and lays the groundwork for future research on the broader contributions of clinical pharmacy services.

We hope these revisions and clarifications address your concerns adequately. We are grateful for your insightful suggestions, which have helped improve the manuscript's scientific rigor and applicability. Please let us know if further adjustments are required.

Warm regards,
Watheq M. Al Setohy

On behalf of all authors

# Point – to – Point reply to additional Journal requirements comments.

1. **Formatting Compliance: The journal requires the manuscript to follow PLOS ONE's style guidelines for formatting, including file naming, as outlined in their templates for the main body, title, authors, and affiliations.**

We have revised the manuscript and modified the format to fully adhere to the journal's formatting requirements, using the provided templates.

2. **Data Availability Statement: The Data Availability Statement must include the contact details (email address or link) for the third party responsible for data requests.**

An email address for requesting the data has been added to the manuscript to ensure compliance with the journal's data availability requirements.

3. **Ethics Statement Placement: The Ethics statement should appear only in the Methods section and must be removed from any other sections of the manuscript.**

The Ethics statement has been moved exclusively to the Methods section of the manuscript and removed from other sections, as per the journal’s guidelines.

4. **References Review: The reference list must be complete and accurate, with no retracted papers cited unless clearly justified and marked as retracted. A citation for the retraction notice must also be included.**

I have thoroughly reviewed and corrected the reference list to ensure it is accurate and complete. No references were deleted or replaced from the originally submitted list, and to the best of my knowledge, no retracted papers are included. However, as part of my reply to reviewer comments to provide additional information on probability estimates derivation and validation, I have added four new references ([35]–[38]) to the manuscript. The new references are as follows:

- **[35]** Cook DJ, Fuller HD, Guyatt GH, Marshall JC, Leasa D, Hall R, et al. Risk factors for gastrointestinal bleeding in critically ill patients. Canadian Critical Care Trials Group. *N Engl J Med.* 1994;330: 377–381. doi:10.1056/NEJM199402103300601
- **[36]** Ben-Menachem T, Fogel R, Patel RV, Touchette M, Zarowitz BJ, Hadzijahic N, et al. Prophylaxis for stress-related gastric hemorrhage in the medical intensive care unit. A randomized, controlled, single-blind study. *Ann Intern Med.* 1994;121: 568–575. doi:10.7326/0003-4819-121-8-199410150-00003
- **[37]** Cook DJ, Griffith LE, Walter SD, Guyatt GH, Meade MO, Heyland DK, et al. The attributable mortality and length of intensive care unit stay of clinically important gastrointestinal bleeding in critically ill patients. *Crit Care.* 2001;5: 368–375. doi:10.1186/cc1071
- **[38]** Tsiotos GG, Mullany CJ, Zietlow S, van Heerden JA. Abdominal complications following cardiac surgery. *Am J Surg.* 1994;167: 553–557. doi:10.1016/0002-9610(94)90096-5
